# Targeting Epigenetic Mechanisms in Vascular Aging

**DOI:** 10.3389/fcvm.2021.806988

**Published:** 2022-01-04

**Authors:** Zhongxiao Lin, Qian Ding, Xinzhi Li, Yuliang Feng, Hao He, Chuoji Huang, YiZhun Zhu

**Affiliations:** ^1^State Key Laboratory of Quality Research in Chinese Medicine and School of Pharmacy, Macau University of Science and Technology, Macao SAR, China; ^2^Key Laboratory of Molecular Target and Clinical Pharmacology and National Key Laboratory of Respiratory Diseases, School of Pharmaceutic Sciences and the Fifth Affiliated Hospital, Guangzhou Medical University, Guangzhou, China; ^3^Nuffield Department of Orthopaedics, Rheumatology and Musculoskeletal Sciences, Botnar Research Centre, University of Oxford, Oxford, United Kingdom; ^4^Shanghai Key Laboratory of Bioactive Small Molecules, Department of Pharmacology, School of Pharmacy, Fudan University, Shanghai, China

**Keywords:** vascular aging, epigenetics regulation, DNA methylation, histone modifications, chromatin architecture

## Abstract

Environment, diseases, lack of exercise, and aged tendency of population have becoming crucial factors that induce vascular aging. Vascular aging is unmodifiable risk factor for diseases like diabetes, hypertension, atherosclerosis, and hyperlipidemia. Effective interventions to combat this vascular function decline is becoming increasingly urgent as the rising hospitalization rate caused by vascular aging-related diseases. Fortunately, recent transformative omics approaches have enabled us to examine vascular aging mechanisms at unprecedented levels and precision, which make our understanding of slowing down or reversing vascular aging become possible. Epigenetic *viz*. DNA methylation, histone modifications, and non-coding RNA-based mechanisms, is a hallmark of vascular aging, its deregulation leads to aberrant transcription changes in tissues. Epigenetics mechanisms by mediating covalent modifications to DNA and histone proteins, consequently, influence the sensitivity and activities of signaling pathways in cells and tissues. A growing body of evidence supports correlations between epigenetic changes and vascular aging. In this article, we will provide a comprehensive overview of epigenetic changes associated with vascular aging based on the recent findings with a focus on molecular mechanisms of action, strategies to reverse epigenetic changes, and future perspectives.

## Introduction

Aging processes are accompanied with the accumulation of degenerative processes and changes in both physiological and functional parameters in mammals. In humans, aging associated vascular diseases constitutes a significant risk to health and the quality of life for individuals. Indeed, approximately 4 million people die from cardiovascular diseases (CVDs) each year in China (1). With the dramatic growth in aged populations around the world, CVDs are one of the biggest global challenges to health care systems.

Undoubtedly, a substantial amount of aging research has focused on finding ways to remove or counteract the loss of biological function in cells and tissues, with the hope of maintaining health or to even extend lifespan. Advances in our understanding of genetics including, “the central dogma,” “heritable traits” and the molecular mechanisms of gene regulation has underpinned breakthroughs in this field and has spawned a growing branch of research known as epigenetics. Epigenetics refers to a phenotype or changes in gene expression caused by mechanisms other than the alteration in a genetic or DNA sequence. These changes result from random events, the impact of environmental factors, diet, and stress, each of which can have significant influence on health and diseases processes in humans (2). Our understanding of the biological processes associated with the aging has advanced in recent years. Indeed, the causes and consequences of aging have been categorized by some researchers into specific “aging phenotypes” grouped by association like for example, oxidative stress and energetics, mitochondrial dysfunction, homeostatic mechanisms, shortening of telomeres, cell senescence, DNA damage, defects in proteostasis, and exhaustion of progenitor cells (3, 4). One common thread linking each of these catagories is that the biological aging process takes many years before it finally translates into structural and functional deterioration. Therefore, to delay aging or, to prevent it from worsening, we first need to understand the fundamental processes that govern these changes.

**Figure 1:**
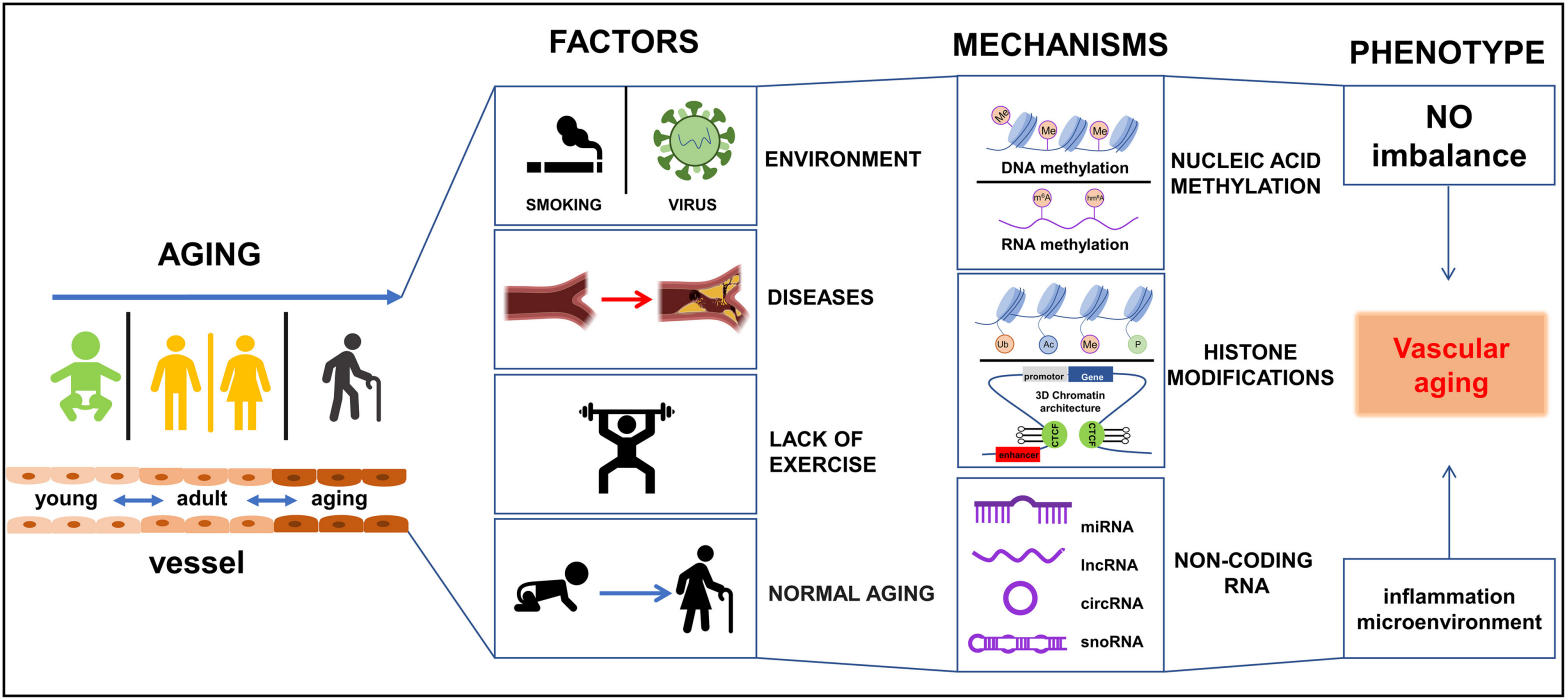
Schematic diagram of epigenetics-mediated vascular aging in cardiovascular system. Multiple factors contribute to vascular aging phenotypes, including environment factors like smoking and virus, various diseases like diabetes and metabolic syndrome, lack of exercise, and normal aging. Epigeneitc modifications of nucleic acid (DNA and RNA) like methylation and demethylation, histone modifications [like methylation, acetylation, ubiquitination, phosphorylation, 3-dimensional (3D) chromatin architecture], and non-coding RNAs, drive vascular aging processes. Ac, acetylation; Me, methylation; P, phosphorylation; Ub, ubiquitination.

During vascular aging dysfunctions in blood vessels, including cellular senescence, vascular remodeling, vascular homeostatic imbalance, inflammation, VSMCs invasion, fibrosis, calcification, the decline in oxygen and nutrient delivery that drives disease severity (5). In each of these scenarios, epigenetic changes have been reported to play important roles in these processes (6). To date, various studies have reported on the mechanisms by which vascular damage occurs during aging, particularly in vascular-related tissues *viz*. ECs, VSMCs, epidermal cells, and extracellular matrix (ECM). Vascular aging provides instructions for selective gene expression, and it is closely related to a variety of vessel-related diseases, such as hypertension, diabetes, atherosclerosis, and hyperlipidemia. Dysregulation in these systems affects DNA modifications, chromatin structure, and gene expression, and is partly responsible for the occurrence and development of vascular aging and age-related diseases in the cardiovascular system (7). Therefore, an understanding of the intrinsic mechanisms of epigenetic regulation in vascular tissues is paramount for the future development of strategies to reduce disease burden in the general population. Currently, epigenetic-mediated aging mechanisms have gained interest from the academic community fueled by advances in omics technologies. These epigenetic mechanisms including DNA methylation (DNAm), RNA methylation (RNAm), histone modifications, and non-coding RNAs (ncRNAs) regulation. In the current review, the pathophysiological changes and roles of epigenetic-mediated vascular aging will be discussed. In addition, advances in our understanding of the underlying molecular mechanisms, and potential therapeutic strategies to manage these changes will be covered. It is hoped this information will assist in the development of novel approaches to diagnosis, treat, and manage vascular-related diseases in humans as summarized in Figure 1.

## Epigenetic Mechanisms in Vascular Aging

### DNA Methylation

The process of DNA methylation (DNAm) has been known since 1950 (8), and a further 17 types of chemical modifications have since been discovered that impact on DNA (9). DNAm acts *via* the attachment of methyl groups, hydroxymethyl, or other moieties to the carbon-5 position in the dinucleotide CpG. CpG islands (regions of DNA with a high G+C content and a high frequency of CpG dinucleotides) usually reside in or adjacent to the transcription start site (TSS). The DNAm status of DNA can therefore influence gene expression (either suppresses or activates) by influencing the interactions of transcription factors (TFs) with target sequences. The addition of methyl groups to DNA is regulated by DNA methyltransferases (DNMTs) including DNMT1, DNMT2, DNMT3A, DNMT3B, DNMT3L. Interestingly, both DNMT2 and DNMT3L are non-canonical family members that lack DNMT activity (10). DNAm can be divided into two main processes *viz*. hypermethylation and hypomethylation. DNA hypermethylation (promotor region) usually leads to the gene transcriptional repression, whereas hypomethylation causes gene transcriptional activation (11). Interestingly, Agha and colleagues recently showed that hypermethylation levels at 52 CpG regions in blood leukocytes can be used as a predictive risk marker for myocardial infarction and coronary heart disease (12). Similarly, in atherosclerotic patients, global DNA hypomethylation was found in peripheral white blood cells, vascular smooth muscle cells (VSMCs), and plaques (13), which means that deleterious genes could be overexpressed, of note, it depends on the balance between hazardous genes and the good ones.

DNA hydroxymethylation is also a crucial component of the epigenetic system, and it is governed by the activities of serine hydroxymethyltransferases (14). Ten-methylcytosine dioxygenase family members 2 (TET2) is one of the serine hydroxymethyltransferase involved in the oxidation of 5-methylcytosine (5-mC) to generate 5-hydroxymethylcytosine (5-hmC). Overexpression of TET2 attenuates intimal hyperplasia (15). DNA modification rarely affects vascular aging through a single enzymatic pathway, but rather alters multiple genes networks. DNMT1, DNMT3A, DNMT3B, TET2, and sensitive components of these networks are crucial as vascular aging targets. Decreased DNMT1 expression occurs in replicative senescent aortic SMCs and correlates with rates of hypomethylation of COL15A1 (over-expressed in the atherosclerotic lesion and localized to the atherosclerotic cap), this linking epigenetic regulation of DNMT1 with SMC phenotypes and prevalence rates of atherosclerosis (16). Epigenetic network including DNMT3A and TET2 are thought to act as tumor suppressor genes *via* their propensity to aid recruitment of histone deacetylases to gene promoters. Interestingly, in the cardiovascular system, reduced activity of DNMT3A and TET2 promotes artery smooth muscle cell proliferation and endothelial cell dysfunction. It is likely that these two genes also play corresponding roles in aging (17). Indeed, DNMT3B together with DNMT3A form a protein complex that interacts with histone deacetylases HDAC1, HDAC2, Sin3A, and the transcriptional suppressor proteins, Rb, TAZ-1, and heterochromatin proteins HP1, SUV39H1. As a result of these interactions, normal levels of DNA methylation and gene silencing are maintained. For example, complex in CpG sites in the promoter of p16(INK4A, an aging marker) leads to downregulation of p16(INK4A) expression (18). Methylation processes also affected by the supply of methyl groups, methylation patterns, folic acid metabolism, and hormone levels. There are still many unknown phenomena in the current research, and many studies need to be further conducted, such as the relationship between random DNA methylation drift in vascular aging. We believe that the methylation regulation is still in the experimental/initial stage, but has attracted the attention of many scholars.

### Histone Modifications

Histone proteins are basic proteins abundant in lysine and arginine residues and include H1, H2A, H2B, H3, and H4. These proteins are responsible for maintaining chromatin structure, mediating dynamic and long-term gene regulation, post-translational mutations, and functional variation. These proteins are subjected to a myriad of modifications including acetylation, methylation, phosphorylation, ubiquitination, butyrylation, formylation, and succinylation. Histones are tightly associated with DNA and altered histone modifications impacts on DNA replication and repair, and the rates of transcription thus impacting on gene expression. Indeed, aberrant histone modifications, for example the dysregulation of histone deacetylase (HDACs), site- specific loss, and gains of heterochromatin are proposed as hallmarks of epigenetic change that can impact on 3D chromatin structure and control gene expression (19). However, many functional changes brought by histone modifications remained largely uncharacterized but research points to a complex regulatory network affected by the spatial structural features and external stress (20). For example, omics approaches reveals that the alteration in the three-dimensional (3D) chromatin architecture caused by histone modification is a crucial epigenetic element important in development, cancer, and aging (21).

Another important class of enzymes are the histone methyltransferases (HMTs) that catalyze histone methylation, typically at lysine and arginine residues present in histone proteins. The mono-methylation of H3K27, H3K9, H4K20, H3K79, and H2BK5 is usually related to gene activation, while that of tri-methylation of H3K27, H3K9, and H3K79 confers inhibition (22).

Histone acetyltransferases (HATs) and histone deacetylases (HDACs) are the most widely studied histone-modifying enzymes in vascular aging. HATs transfer acetyl group onto histone proteins, which in turn neutralizes the charge of histone, and weakens its interaction with DNA. Acetylation of H3K122 and H3K64 breaks the interaction between histone tails on adjacent nucleosomes and loosens the connection between nucleosomes (23). Histone acetylation can be identified by the bromodomain of TATA-binding protein-associated factor1 (TAF1), which attracts various TFs, and promotes transcription. HDACs have opposite effects to that of HATs and a dynamic balance between the activities of HATs and HDACs governs gene expression. In addition, histone phosphorylation plays a role in altering chromatin architecture like for example, the phosphorylation of H3T118 that enhances DNA accessibility, nucleosome mobility, and nucleosome disassembly (24). Other modifications including histone H2A ubiquitination reinforce nucleosome stability (25), and the hyper-ubiquitination of this target is considered an aging associated biomarker (26). In addition, chromatin architecture is also a crucial regulator. Chromatin is composed of different structural units, from the compartment, topologically associated domains to loops, and all these units have their characteristics and regulate chromatin function and gene expression (27). Indeed, the CCCTC-binding factor (CTCF) architectural protein can be up-regulated in pluripotent stem cells (iPSCs) helping in the formation of compact chromatin loops. However, reductions in CTCF disrupts the loop structure and promotes the overexpression of the p16 (INK4a), an aging-induced gene (28). Histone modification in the cardiovascular system is an active field of research with growing evidence implicating a role of HDACs in the regulation of vessel homeostasis. Indeed, HDAC4 regulates vascular inflammation *via* activation of autophagy in endothelial cells (29) and the deletion of HDAC9 promotes inflammation resolution and reverse cholesterol transport in atherosclerosis and coronary heart diseases (30, 31). In addition, elevation of acetyltransferase p300 accelerates aging (32). Histone H3 lysine 4 (H3K4) methyltransferase Smyd3 (SET- and MYND- domain-containing proteins) directly binds to the promoter region of Cdkn1a (coding for p21). This interaction causes an increase in H3K4me3 and leads to the activation of p21 giving rise to SASP. These SASPs can be reversed by the Smyd3-specific inhibitor EPZ031686 (33), but enhanced when the Hsp90α binds to Smyd3 (34). Collectively, the available research clearly shows that modification of histone proteins, either by methylation, acetylation, or ubiquitination, has downstream impacts on genes associated with vascular aging. Other systems include the Smyd family proteins that methylate H3K9, H3K27, H3K36, H4K20, and H3K79 (35). Interestingly, tri-methylation of H3K27 and H3K36 is associated with accelerated epigenetic aging in humans (36). These impacts are linked to changes in 3' untranslated region (UTR) length and the methyltransferase (MET-1) activities (37, 38). H3K27 is also a target for histone demethylase Jumonji domain-containing protein 3 (JMJD3) regulates vascular neointimal hyperplasia by mediating H3K27 tri-methylation (39). These modifications enlighten us that modifications at variant sites impact gene function in different styles.

In addition, NAD+ -dependent processes are critical for maintaining tissue and metabolic homeostasis relative to vascular aging. Diminished tissue concentrations of NAD+ leads to downregulation of SIRT family expression. The SIRT family proteins are positively associated with longevity and roles for SIRT1 in reducing vascular senescence, inflammation, DNA damage, and atherosclerosis are widely reported (40). Here we focused on SIRT6 and SIRT3 in vascular function. SIRT3 enhances the expression of the blood pressure regulator GATA5 (GATA-binding protein 5). The endothelial specific loss of GATA5 causes vascular endothelial dysfunction *via* the inhibition of transcriptional repressor Nkx3 mediated by deacetylation of histone H3K9. The SIRT6/GATA5 signaling pathways could be a way to reduce endothelial senescence and apoptosis (41). Another anti-atherosclerotic mechanism linked to SIRT6 is in the maintenance of endothelial function *via* its propensity to deacetylate H3K9 in the promoter region of the pro-atherogenic target TNFSF4 (42). Other components associated with vascular function include the p66^shc^, an epigenetic factor associated with diabetes-induced vascular senescence *via* its ability to inactivation miR-34a (43) and SIRT3 (44). SIRT3 is mainly expressed in mitochondria, while SIRT6 in the nucleus this fact highlighting the challenging nature of developing drugs targeting these proteins.

### Non-coding RNAs

NcRNAs have been shown to be crucial for the maintenance of vascular function by regulating nuclear transcription and gene translation in cytoplasm. NcRNAs are divided into small or short ncRNAs [(smaller than 200 nucleotides (nt)], long ncRNAs (lncRNAs, longer than 200 nt), microRNAs (miRNAs, 21–25 nt) belonging to the small class of RNAs, and circular RNAs (circ RNAs, 300–500 nt) pertain to lncRNAs. To date, various classes of ncRNAs have been shown to influence inflammation, senescence, cellular function, and differentiation. For example, miR-92a blocks endothelial proliferation while miR-24 promotes vascular stress (45). miRNA bind to mRNA promoting degradation or the inhibition of translation. CircRNAs interact with miRNAs to form a circRNA-miRNA-mRNA loop regulatory unit, and crosstalk between components of this system plays a crucial role in the development of vascular diseases by altering pathways like cell adhesion, immune response, and regulation of cell adhesion (46). LncRNAs are often associated with homologous DNA and RNA sequences exerting their regulatory role in cells and tissues (47). Small nucleolar RNA (Sno-ncRNAs, 60–300 nt) are a class of nuclear-enriched intron-derived ncRNAs lacking 5' caps and 3' poly(A) tails. These molecules are widely expressed in tissues and are composed of two types, box C/D and box H/ACA snoRNAs; these types respond to 2'-O-ribose methylation and pseudouridylation, respectively (48). SnoRNAs are involved in oxidative stress responses and may therefore be important in regulate aging processes (49). MiR-22 and miR-128 possess therapeutic and prognostic potential as a novel target to treat post-infarct and aged-VSMC remodeling (50, 51). This ncRNA controls VSMC phenotypes and injury-induced arterial remodeling by modulating multiple genes including methyl-CpG binding protein 2 (MeCP2), SIRT1, HDAC4, and EVI1. Other epigenetic modifications including the hypomethylation of the lncRNA H19 (H19) promoter region can lead to the silence of NOTCH1. In this cascade, silencing of NOTCH1 prevents the recruitment of p53 to the NOTCH1 promoter. In turn, this leads to calcific aortic valve disease, endothelial injury, matrix remodeling, angiogenesis, and calcification (52). CircRNAs and snoRNAs are also regarded as critical components of ncRNAs. For example, circ ANRIL levels correlate with increased atherosclerosis risk (53). Moreover, the target SnoRNA U17 regulates cellular cholesterol trafficking (54). However, the molecular mechanism of action for each of these regulates is still unclear and warrants further investigation.

### Factors Link to Epigenetic Mechanisms

The epigenetic network encompasses a wide range of biological reactions, and it is likely that even with current technologies, researchers only capture a fraction of the changes that occurs in cells and tissues. Here we hold the opinion that some factors linked to epigenetic systems associated with vascular aging is of importance, as summarized in Table 1.

**Table 1:** Vascular aging-associated epigenetic alterations and its rationale.

**Gene/protein**	**Function**	**Aberration**	**Suggested mechanisms**	**Study model**	**Tissue/cell type**	**Outlook**
**DNA and RNA modification**						
DNMT1	DNMT	LOF/OE	Gene-specific DNA methylation	*in vitro*	Coronary Plaque, ECs, Aortic SMCs	Inhibitors have been in clinical use, more small molecular compounds are under developing
DNMT3A	DNMT	LOF/OE	Gene-specific DNA methylation	*in vitro*		-
DNMT3B	DNMT	LOF/OE	Gene-specific DNA methylation	*in vitro*		-
METTL3	RNA methylation	LOF	Gene-specific RNA methylation	*in vitro*	Human, mouse aortic VSMCs	Alleviates cellular senescence
TET2	DNA hydroxylase	LOF	Gene-specific DNA methylation, increase 5-Mc and decrease 5-hmC	*in vitro and in vivo*	SMCs	Potential DNMT inhibitor
**Histone modification**						
HDAC4	HDAC	OE	Regulates vascular inflammation	*in vitro and in vivo*	VECs	Inflammation inhibition
HDAC9	HDAC	OE	HDAC9 deficiency promotes inflammation resolution	*in vivo*	Human	Block atherosclerosis progression
P300	HAT	OE	Elevated P300 leads to vascular injury	*in vitro and in vivo*	Human/cardio-vascular system	Inhibition P300 relieves cardiovascular aging
SMYD3	HMT	OE	SMYD3 increases p21 and promote cellular senescence	*in vitro and in vivo*	VECs	Prohibit SMYD3 rescue endothelial senescence
JMJD3	HDM	OE	Attenuates vascular remodeling	*in vitro and in vivo*	VSMCs	Inhibit neointima formation after injury
SIRT1	HDAC	LOF	SIRT1 homeostasis reduce vascular senescence, inflammation, DNA damage	*in vitro and in vivo*	VECs, VSMCs	Positively associated with longevity
SIRT3	HDAC	LOF	Modulates age-associated mitochondrial biology and function	*in vivo*	Human, mouse aortic	Diabetes-induced vascular senescence
SIRT6	HDAC	LOF	Prevents endothelial injury	*in vivo*	VECs	Anti-atherosclerotic
**Non-coding RNAs**						
miR-22	miRNA	LOF	Promotes arterial remodeling by mediating MeCP2, SIRT1, HDAC4, and EVI1	*in vivo*	VSMCs	A potential therapeutic agent in coronary atherosclerosis
miR-128	miRNA	OE	Targeting KLF4 and regulate VSMCs phenotypic switch	*in vitro and in vivo*	VSMCs	Inhibition of miR-128 is beneficial to cardiovascular
miR-214	miRNA	OE	Preventes Ang II-induced periaortic fibrosis	*in vitro and in vivo*	Human, mouse, rat aortic and plasma	A target to prevent vascular stiffening
H19	lncRNA	OE	H19 silenced NOTCH1 by preventing the recruitment of p53 to its promoter	*in vitro and in vivo*	Human, mouse calcific aortic valve	-
ANRIL	circRNA	LOF	Promotion of ASVD by modulating INK4/ARF gene transcription	*in vitro*	HUVEC and other cells	A possible ASVD diagnostic marker
U17	SnoRNA	LOF	Regulates cellular cholesterol homeostasis	*in vitro*	CHO-K1 cells and NIH3T3 cells	-
**ROS-mediate epigenetic changes**						
MOF	HAT	OE	Modify the chromatin structure surrounding the NOX promoters	*in vitro and in vivo*	Macrophages and cardiomyocytes	-
SIRT3	HDAC	LOF	Enhance the expression of SOD2	*in vivo*	Human, mouse, and arterioles	-
**Epigenetic in Integrin family**						
Integrin β1	membrane receptor	LOF	Hypermethylation status of St6gal1 restrains Integrin β1 and actives adipogenesis	*in vitro and in vivo*	Mouse, 3T3-L1 cells	A possible suppressor targeting St6gal1 hypomethylation
Integrin αvβ3	membrane receptor	LOF	Influence angiogenesis and relate to HDAC5	*in vitro*	HUVECs	-
Integrin Gα13	membrane receptor	LOF	Suppress the phosphorylation of YAP/ TAZ-JNK and reduces plaque formation	*in vitro and in vivo*	Mouse carotid artery, HUVECs	-
**Epigenetic in toll like receptor family**						
TLR2	Toll like receptor	OE	Through the association with epigenetic markers, DNMT1, HDAC1, SUV39H1, TET2, and promote NF-κB	*in vitro and in vivo*	Coronary artery disease	An inflammation inhibition target
TLR4		OE				
TLR9	Toll like receptor	OE	Mediate immune responses by recognizing CpG-motif of ODN	*in vitro*	Raw264.7 macrophage	-
**Epigenetic in Ca**^**2+**^ **family**						
TRPM7	calcium channel	OE	Promotes histones H3K9, H3K27 acetylation, H3 phosphorylation107, and chromatin covalent modifications	*in vitro and in vivo*	Mouse, ESCs	-
LTCCs	calcium channel	OE	Negatively regulating by miR-328 in hypertensive rats	*in vitro*	VSMCs	-
**Epigenetic in hormone family**						
Testosterone	hormone	LOF	Ameliorates vascular aging *via* the GAS6/AXL signaling pathway, GAS6 can be activated by lncRNA SWINGN.	*in vivo*	Mouse, carotid artery	-
Progesterone	hormone	LOF	Changing the responses of NO handling *via* a H3K27ac- and H3K27me3-dependent manner	*in vitro*	Endometrial stromal cells and decidualized cells	-
Estrogen	hormone	LOF	Activates SIRT1, H3 acetylation, inhibits VSMCs proliferation, increases endothelial migration	*in vitro and in vivo*	Human, rat carotid artery	-
**Epigenetic in MMP family**						
MMP2	ECM enzyme	OE	SIRT1 negatively regulates aortic MMP2 and MMP9 expression, and blocks medial degeneration	*in vitro and in vivo*	Mouse, thoracic aorta SMCs	-
MMP3	ECM enzyme	OE	Regulated by H3K9me2	*in vitro and in vivo*	Mouse, atherosclerotic lesions, VSMCs	-
MMP9	ECM enzyme	OE	Is a driving factor in macrophage-dependent inflammation	*in vitro and in vivo*	Mouse, atherosclerotic lesions, VSMCs	Inhibition of MMP is a target to reduce macrophage polarization
MMP12	ECM enzyme	OE	Regulated by H3K9me2	*in vitro and in vivo*	Mouse, atherosclerotic lesions, VSMCs	-

*HAT, histone acetyltransferase; HMT, histone methyltransferase; HDM, histone demethylase; DNMT, DNA methyltransferase; HDAC, histone deacetylase; VECs, vascular endothelial cells; ASVD, Atherosclerotic vascular disease; NOX, NADPH oxidase; HUVEC, Human Umbilical Vein Endothelial Cells; VSMCs, vascular smooth muscle cells; LOF, loss of function; OE, overexpression; ESCs, embryonic stem cells; ECM, extracellular matrix*.

#### ROS

ROS (reactive oxygen species) regulates vascular aging by impairing the function of endothelial nitric oxide synthesis (eNOS). Two regulatory mechanisms are involved. The first involves epigenetic mechanisms impacting on ROS accumulation like for example, NADPH oxidase (NOX). NOX is a source of ROS that is significantly increased in vascular aging. It is regulated by the transcription factor megakaryocytic leukemia 1 (MKL1). MKL1 recruits the histone acetyltransferase (MOF) to modify the chromatin structure surrounding NOX promoters, and this leads to the enhancement of NOX catalytic activity. Increased NOX activity drives rates of ROS accumulation in tissues (55). The second mechanism involved in ROS production involves the epigenetic processes that abolish ROS production or damage. In this regard, ROS scavenging enzymes like superoxide dismutase 2 (SOD2) are critical. Excessive ROS promotes increased levels of methylation at the SOD2 promoter region causing transcriptional silencing. Moreover, expression levels of SOD2 can be enhanced by SIRT3 (56). We speculate that SOD2 activator molecules would be useful therapeutic tools to manipulate SOD levels on tissues and deserves investigation. Indeed, SOD can reduce excess ROS and contributes to facilitating femoral artery endothelial function (57).

ECs synthesized NO at discrete concentrations whereby it suppresses VSMC relaxation and maintains low rates of cellular proliferation. However, under physiological conditions of excessive ROS production rates of NO production become dysregulated, promoting arterial stiffness, collagen synthesis, intimal hyperplasia, and apoptosis. Interestingly, treating with the NOX1/4 inhibitor, GKT137831, dampens ROS generation and is protective in this setting (58). Furthermore, endogenous ROS contribute to DNA repair and impairment, and this regulates cell development and differentiation in a PI3K/Akt-dependent manner (59). Aberrant activation of AKT disturbs rates of cellular proliferation, cell survival, and metabolic homeostasis by altering DNA methylation and histone modifications; processes impacted by the activities of Foxo1/3, p53/21-dependent pathways that drives rates of cellular senescence (60, 61).

#### Integrin Family

Integrins are a family of membrane receptors that are expressed widely on the surface of cell membranes. These proteins mediate cellular adhesion to the extracellular matrix and function by allowing cells to sense changes to their localized environment. Integrins are crucial components with roles in thrombosis, leukocyte infiltration, VSMC aggregation, cell migration, ECM deposition, and in the vascular phenotype switch (62). Interestingly, integrin β1, has recently been found to mediate capillary aging and angiogenesis. Indeed, loss of endothelial integrin β1 leads to endothelial cell differentiation defects, cell adhesion suppression, reduced capacity to aid cellular migration, and survival inhibition during angiogenesis. In contrast, upregulation of integrin β1 results in SASP (63, 64). In terms of epigenetic mechanism, integrin β1 expression is regulated by β-galactoside α2,6-sialyltransferase-1 (St6gal1) DNAm (65). Other integrin proteins like integrin αvβ3 promote coronary arteriolar dilation and angiogenesis in a HDAC5-dependent manner (66). Furthermore, integrin Gα13 is atheroprotective (67).

#### Toll-Like Receptor (TLR) Family

TLRs can be activated during physiological and pathological aging, and induction results in a robust inflammatory response. For example, endothelial TLR4 drives lesion formation and causes stroke and seizure (68). TLR4 expression negatively correlates with regulatory factor X1 (RFX1). RFX1 reportedly increases rates of methylation of histone H3K9, and decreased levels of H3 and H4 acetylation in the TLR4 promoter *via* the involvement of DNMT1, HDAC1, and histone-lysine N-methyltransferase SUV39H1 (SUV39H1), respectively. In addition, TET2 impacts on TLR4 *via* the NF-κB p65 pathway (69). Dimerization of TLR2 and 4 induces the activation of the NLRP3-inflammasome resulting in diminished endothelial regeneration (70). TLR9 recognized CpG-motif of oligodeoxynucleotides (ODN) and elicited immune responses that lead to the induction of apoptosis (71). Clearly, given the intimate role that TLRs play in vascular damage and aging, these proteins could be useful drug targets.

#### Ca^2+^ Channels

Ion channels are involved in signaling networks and homeostatic regulation. Genetic variants coding for ion channels and epigenetics targets can impact on structural disorganization and functional defects. Ca^2+^ is a secondary messenger needed for proper cell function. The Ca^2+^ channel superfamily includes transient receptor potential (TRP) family of proteins namely, TRPC, TRPV, TRPM, TRPA, TRPML, TRPP, the ORAI family, and Ca^2+^-activated K^+^ (K_Ca_) channel family (72). Combined these channels are critically important in the regulation of calcium signaling systems in cells and tissues. Some Ca^2+^ channels play a direct role in vascular aging. For example, TRPCs are non-selective cation channels permeable to calcium ions. Among them, TRPM7 affects histones H3K9, H3K27 acetylation, H3 phosphorylation, and chromatin covalent modifications (73). Increased acetylation status of H3K27 is associated with vascular aging. Tentative links between the Orai family members and vascular aging have also been proposed in recent times (74), however, the epigenetic mechanisms involved in their regulation are still unknown. Other calcium channels including the Ca^2+^-activated K^+^ (K_Ca_) channels that act as transducers, that respond to elevated intracellular calcium ([Ca^2+^]i) signals causing hyperpolarization of V_m_ and decreased vascular resistance thereby enhancing blood flow. K_Ca_ channels facilitate Ca^2+^ influx into cells *via* non-selective cation channels that can stimulate increased synthesis of nitric oxide (NO) (75). Dysregulation of Ca^2+^ handling and the activities of K_Ca_ channels precipitate reductions in NO levels, leading to increased vascular stiffness, and eventually vascular aging. Lastly, dysfunction of L-type voltage-gated Ca^2+^ channels (LTCCs) exert an epigenetic influence by negatively regulating levels of miR-328 in artery-derived VSMCs (76).

#### Hormone

After entering adolescence, males and females produce germ cells and secrete sex hormones (the sex steroid estrogens, androgens, and progesterone). Sex hormones promote the development of mature reproductive organs, reproductive function, and the secretion of sex hormones and regulatory systems. Mounting evidences have shown that the incidence of vascular diseases in elder male and postmenopausal female were age- and gender- specific. Both estradiol and testosterone, which modulate female and male endothelial function, typically fall in post-menopause females and elderly males. The low level of these two hormones correlates with vascular aging in women and vasomotor instability in male (77). The sex hormone cytosolic/nuclear receptors for estrogen, progesterone, and testosterone have been identified, and shown to function in vascular endothelium and smooth muscle cells signaling networks (78). Indeed, it is likely that these hormones are involved in vascular cell proliferation, development, and in cellular senescence. For example, testosterone ameliorates vascular aging *via* the GAS6/AXL signaling pathway (79). GAS6 signaling is reported to increase NO bioavailability *via* the androgen receptor-mediated activation of endothelial NO synthase (eNOS) (80). Testosterone may also function *via* lncRNA-SWI/SNF complex crosstalk (81). Interestingly, while therapeutic doses of testosterone can benefit elderly individuals, excessive circulatory testosterone has a detrimental effect. This negative impact of testosterone appears to be due to impaired endothelial progenitor cells function partly linked to the hypermethylation on the estrogen receptor β's promoter. Similarly, the administration of progesterone in postmenopausal women have been widely used therapeutically for the existence of epigenetic phenomena that progesterone influences the responses of cells to NO handling *via* a H3K27ac- and H3K27me3 dependent process (82, 83). Similarly, estrogen acts on vascular aging by activating SIRT1, H3 acetylation, and promotes miR-203 expression. These changes inhibiting VSMCs proliferation and promoting miR-126-3p expression that results in endothelial migration (84, 85). It is feasible that the activation of SIRT1 by estrogen partly explains why female have a higher life expectancy than that of males.

#### MMP Family

Metalloproteinases are a group of proteolytic enzymes that are involved in the metabolism of the extracellular matrix. These enzymes play essential roles in angiogenesis, wound healing, and fibrosis. Interestingly, overexpression of SIRT1 resulted in lower MMP2 expression in VSMCs and this correlates with the deacetylating of histone H3 lysine 9 (H3K9) sites within the MMP2 promoter (86). In addition, metalloproteinase 9 (MMP9) is involved in the decomposition of extracellular matrix and is essential for driving macrophage-dependent inflammation in the vascular system (87). MMP9 levels are regulated by TET2, miR-212, miR-132, and the long non-coding RNA, TETILA (88, 89). H3 lysine 9 di-methylation (H3K9me2), is a repressive epigenetic marker and the levels of which are often reduced in atherosclerotic lesions. H3K9me2 is down-regulation in the promotor regions of MMP3, MMP9, and MMP12 in VSMCs of arteries. Clearly, these three proteins play important roles in controlling VSMCs response to vascular inflammation.

#### RNA Methylation

In the 1970s, scientists discovered that RNA methylation can also occur in cells and tissues and that these modifications are now known to be crucial epigenetic regulators. At present, more than 160 RNA modifications have been identified, including N6-methyladenosine (m6A), 5-methylcytosine (m5C), N7-methylguanosine (m7G), and so on (90). Among these modifications, m6A is the most abundant mRNA modification in eukaryotes, which is mediated by methyltransferase like proteins namely METTL3, METTL14, WTAP (91), and this modification can be reversed by demethylase like FTO, ALKBH5. Besides, m6A binding proteins like YTHDF1/2/3, YTHDC1/2 also take part in the dynamic reversible m6A modification. METTL3 alleviates human mesenchymal stem cell senescence through m6A modification-dependent stabilization of the MIS12 transcript (92), the overexpression of METTL3 coupled with METTL14 promoted SASP-related cytokines (such as CXCL1, CXCL3, CXCL5, CXCL6, IL1α, IL1β, and IL6) releasing (93). The loss of FTO was also reported to antagonize the vascular dysfunction by improving the insulin sensitivity in obesity mice (94). These experimental results showed that RNA methylation is vital in regulating vascular aging processes.

## Mediators in Vascular Aging

### Environmental Factors

Life expectancy is increasing due to advances in the clinical management of diseases, and due to our understanding of lifestyle factors that negatively impact on health. Numerous environmental factors including toxins, heavy metal ions, pollutants, gases, infections, smoking, and alcohol consumption are known to impact on vascular function and influence gene expression. In addition, some epigenetic alterations are heritable like for example, a parental high-fat diet that renders offspring more susceptible to developing obesity and diabetes (95). Of the known environmental drivers of cardiovascular diseases, smoking and microorganism infection are the most widely studied:

#### Smoking

**Figure 2:**
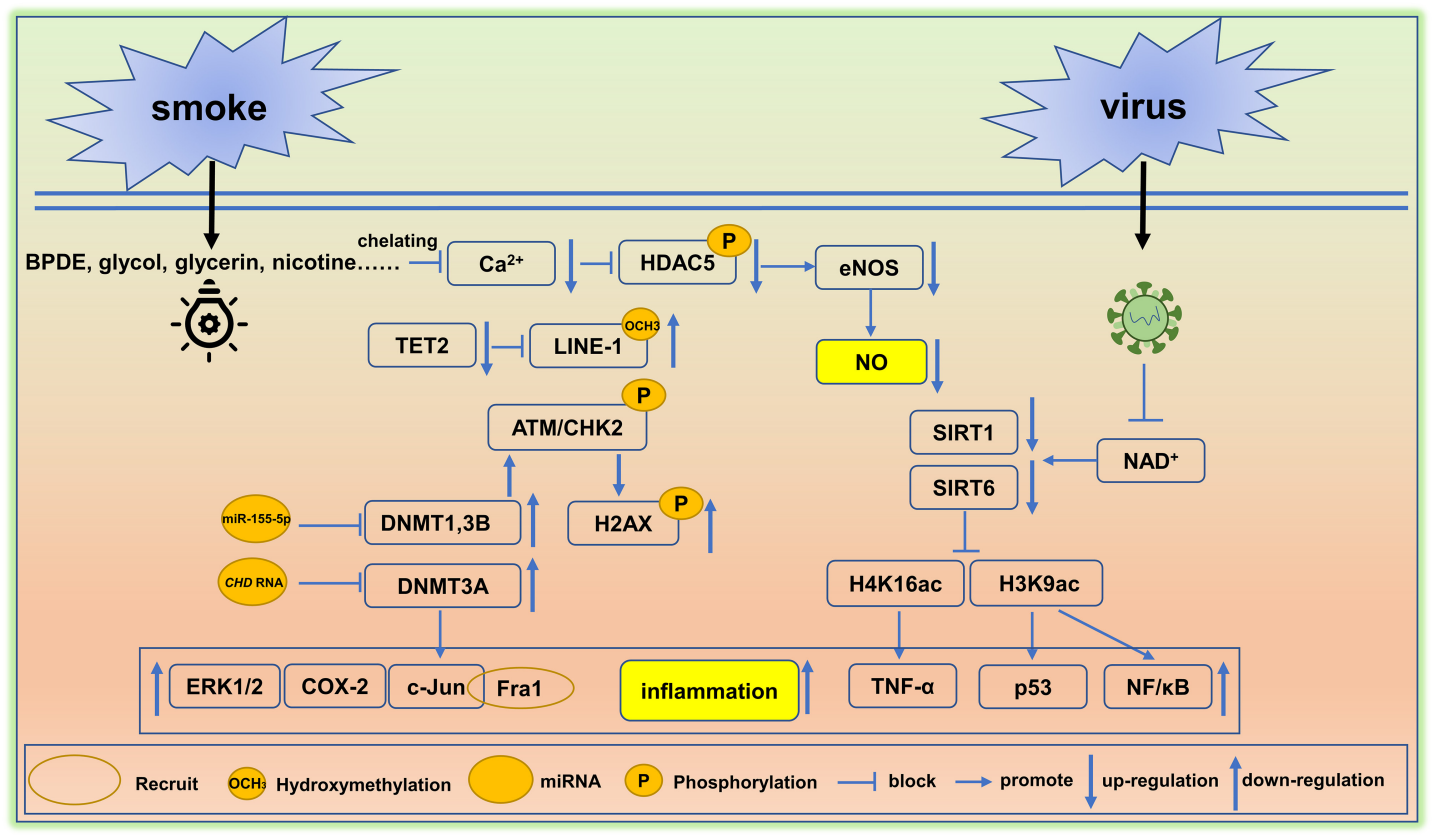
Environment-mediated vascular aging. Smoking and virus are two factors that cause epigenetic alterations in cells and tissues and induce vascular aging. BPDE, Benzo[a]pyrene diol epoxide, DNMT, DNA methyltransferase, eNOS, endothelial NO synthase, TET2, ten-methylcytosine- dioxygenase 2, SIRT, Sirtuin. A detail of these epigenetic pathway is elaborated in section environmental factors.

The adverse effects of smoking on human health have been known for thousands of years and this habit remains one of the leading drivers of premature death and disability worldwide. Tobacco use and its chemical constituents are associated with causing cellular damage including rates of apoptosis, cell cycle arrest, DNA damage, ER stress, and oxidative stress in vascular endothelial cells (96). Moreover, as shown in Figure 2, smoking hastens vascular aging by affecting multiple biochemical pathways. Indeed, the reactive metabolite benzo[a]pyrene diolepoxide (BPDE) causes the accumulation of DNA adducts, that can block the formation of the DNA replication forks in mammalian cells. Persistent blockage of the replication fork by bulky lesions causes DNA double-strand breaks. In turn, these strand breaks elicit histone H2AX phosphorylation (γH2AX, a marker of DNA double-strand breaks) and ataxia telangiectasia-mutated kinase (ATM)/CHK2-mediated events, and this is often seen in aging and in some cancers (97). Furthermore, reductions in DNMT1 and DNMT3B activities protect aging cells from BPDE-induced DNA damage *via* the inhibition of H2AX by ataxia telangiectasia-mutated kinase phosphorylation (98). BPDE also suppresses retinoic acid receptor-β2 (RAR-β_2_) expression by promoting DNMT 3A interactions with the promotor region of RAR-β2. This interaction causes the increased expression of c-Jun, extracellular signal-regulated protein kinases 1/2 (ERK1/2), and cyclooxygenase-2 (COX-2) (99). Of these targets, c-Jun is reported to associate with Fos-related antigen 1 (Fra1), allowing for its binding to the promoter region of Cdkn1a (coding for p21) and Cdkn2a (coding for p16), and this triggers the senescence-related phenotypes, often seen in many cardiovascular disorders and in vascular senescence (100).

Decreased rates of hydroxymethylation in DNA correlates with smoking status. Cigarette smoke ingredients like propylene glycol, glycerin, additives, and nicotine trigger the hypomethylation of LINE-1 repeat elements by reducing global levels of DNA hydroxymethylation. Hypomethylation is typically caused by reductions in TET2 (101), leading to the overexpression of the LINE-1 gene (an aging biomarker) (102). Moreover, TET2 down-regulation is linked with arterial hypertension and immune activation (103, 104).

A study of 20 young healthy smokers including 10 males and 10 females was employed to study the alterations in microRNA signatures. The results showed that miR-29b (associated with aortic aneurysm and fibrosis) was significantly up-regulated and miR-223 is downregulated (105), the loss of miR-223 could lead to severe coronary artery pathology through a miR-223/PDGFRβ vascular smooth muscle cell axis (106). In endothelial cells, the miR-155 inhibition rescued the deleterious effects of cigarette smoke condensate on endothelial-mediated vascular relaxation and oxidative stress (107). Moreover, ncRNAs are critical to the regulation of transcription and in conjunction with DNA methylation rates can influence RNA–DNMT interactions. Indeed, it has been reported that *NUP153, EF1a*, and *CHD* RNA are bound to DNMT3A, yet only *CHD* RNA is capable of inhibiting the catalytic function of DNMT3A (108). These findings are similar to the inhibition of DNMT1 by miR-155-5p (109), suggesting that *CHD* RNA and miR-155-5p are potential therapeutic targets that could be used to restore DNMT3A, DNMT1 (107).

In other studies, the histone deacetylase SIRTUIN1 (SIRT1), which acts as a longevity gene and plays an instrumental role in cell cycle progression, energy metabolism, and aging (110). SIRT1 is reduced by smoking, resulting in an imbalance of downstream histone modifications, like H1K26, H3K9, H3K14, H3K56, and H4K16 (111). Under normal circumstances, SIRT1 binds to the promoter of p53 (a proapoptotic gene) decreasing H3K9 acetylation, and thereby inhibiting p53 gene transcription (112). A similar mechanism has been reported in the suppression of the NF-kappaB gene (113). Smoking clearly disturbs patterns of histone modification and this correlates with a vascular aging phenotype, inflammation, and apoptosis. In addition, smoking causes increased blood pressure leading to elevated fluid shear stress. This in turn, inhibits Ca^2+^ channel signaling, reduces eNOS, and blocks HDAC5 phosphorylation (114).

#### Microorganism Infections

Microorganism infections contribute to vascular aging. For example, the hypercoagulable and hyperinflammatory states caused by the viral infections make vascular endothelial cells more susceptible to infection, resulting in endothelialitis (115). Persistent inflammation promotes the accumulation of toxic metabolites and reactive oxygen species, and further leads to genetic and epigenetic alterations. In this setting, DNA hypermethylation usually activates the senescence- associated secretory phenotype (SASP) (116). Some virus possesses an ADP-ribosylhydrolase that can deplete NAD^+^, and causes a decline in the activities of NAD^+^-dependent lysine deacetylases like the SIRT family proteins, SIRT1 and SIRT6 (117, 118). Loss of SIRT1 attenuates its deacetylation function on the target proteins H4K16 located in the TNF-α promotor region; this leads to increased TNF-α levels, a widely known proinflammatory mediator. Moreover, dramatic reduction in claudin-1 and vascular endothelial-cadherin inactivate DNA repair mechanisms and increase the rates of vascular inflammation and senescence (119). Importantly, TNF-α and IFN-γ triggered inflammation, causing cell death, tissue damage, and mortality (120). In a similar manner to SIRT1, SIRT6 attenuates inflammation *via* reducing NF-κB signaling following the deacetylation of the target H3K9 (113). Based on the available evidence, moderate activation of SIRT1 and SIRT6 could be used to dampen rates of vascular inflammation and could have potential in reducing rates of vascular aging in humans. A summary of environment factors linked to vascular aging are shown in Figure 2.

### Diseases

A wide range of epigenetic alterations has been discovered that are associated with the diseases-induced vascular aging phenotype (121). T-cell–derived miR-214 facilitates perivascular fibrosis in hypertension (122), nuclear miR-320 promotes lipid accumulation (hyperlipidemia) in the heart (123), and genome-wide DNA methylation profiling has discovered significant differences in promoter CpG islands in genes like HIF3A, CPT1A, CD38, PHGDH, ABCG1, SREBF1, CPT1A, PDX1 (124). In addition, the HDAC inhibitor trichostatin A, is reported to reduce atherosclerosis in ApoE-deficient mice, suggesting an important role of epigenetic modifications in the cardiovascular system (125). Other researchers have shown that the histone methyltransferases, SET7 and SET 9, and various histone deacetylases (HDAC4, HDAC7, HDAC10, and KMT2D), along with ncRNA, miRNA-199a-3p, miRNA 34a, circ HIPK3, and circ ZNF609 are linked with vascular dysfunction (126, 127).

### Lack of Exercise

Exercise is an exogenous stimulus that influences cellular metabolism by altering the expression of enzymes and proteins involved in numerous metabolic pathways involved in energy production. A lack of physical activity or a sedentary lifestyle can increase the risk of vascular aging disease, such as arterial stiffening and hypertension (128). However, moderate rates of exercise are seen as beneficial since this makes the myocardial systole more powerful, increases stroke output, enlarges the coronary artery diameter, improves the heart's blood supply, enhances the elasticity of systemic blood vessels, and delays arteriosclerosis. The epigenetic mechanism of exercise in reducing vascular aging will be illustrated in section Health lifestyle.

### Normal AGING

In addition to accelerating aging (above three mediators), the prevalence of vascular diseases is also on the increase as the number of elderly people is increasing worldwide. Even in the absence of overt injury, structural and functional changes occur in vessels as they age. Physiological functions, such as antioxidant enzyme activity, muscle composition, inflammatory factors expression, thickening of the intima, and changes in metabolic enzyme activities rise during normal aging. These age-related vascular changes often accompany or even precede CVD development. Over time, normal aging increases transcriptional noise and stochastic effects in tissues and drives dysregulation of genetic and epigenetic control in the ECM (129). Phenotypically, the aging vessels begin to show moderate increases in the level of peripheral resistance, increased lumen diameter, and changes to the media thickness in small-sized muscular arteries. No accompanying changes in the media-to-lumen ratio occur with this indicating that it is an outward hypertrophic remodeling. Currently, the epigenetic mechanisms that slow down normal vascular aging are usually associated with regulating the daily environment, exercise, and reducing the incidence of disease, which mediate alterations on DNA methylation, histone modification, and ncRNA changes.

## The Epigenetic Intervention Strategies of Vascular Aging

**Figure 3:**
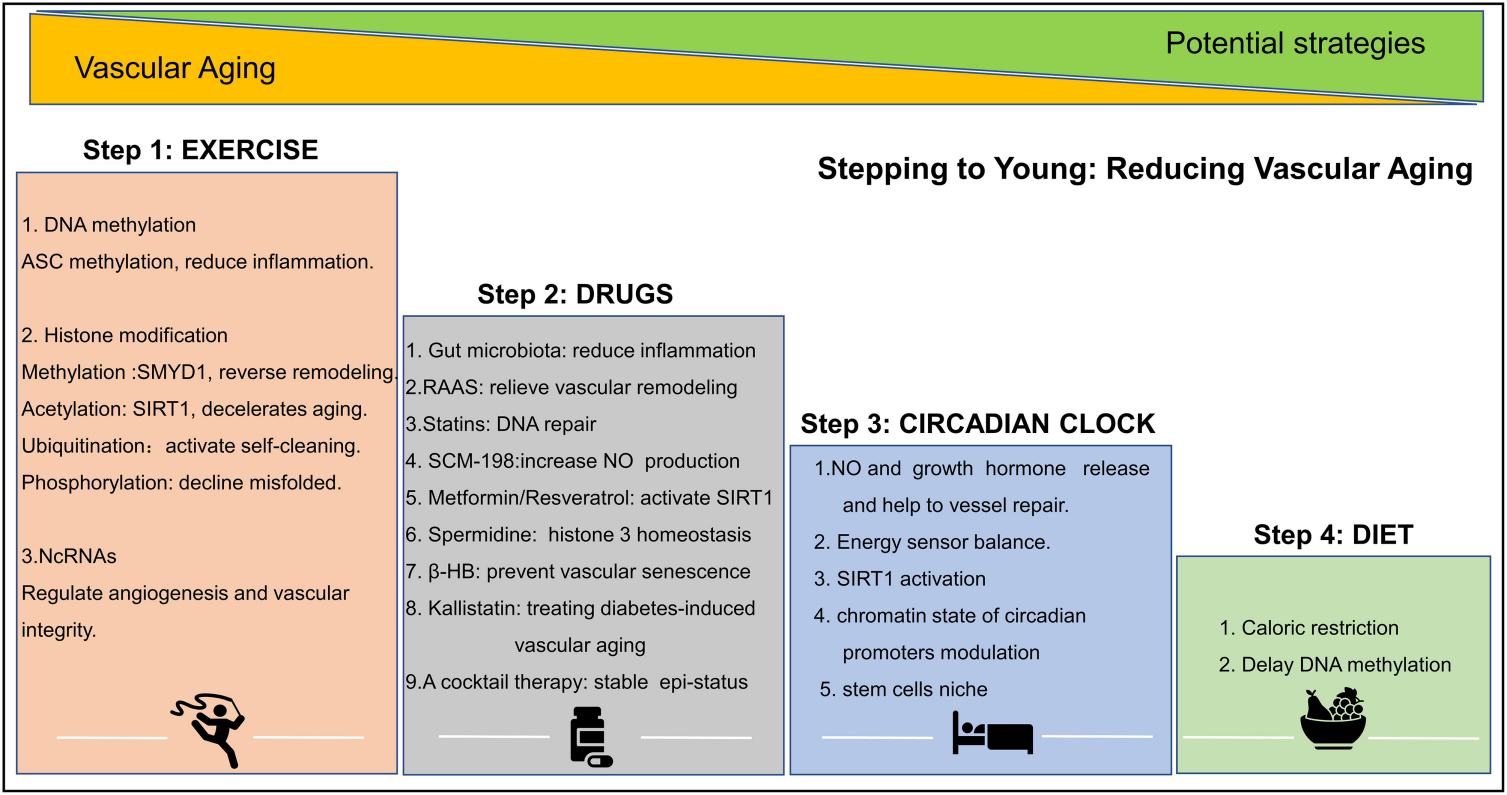
Epigenetic strategies to reduce vascular aging. Exercise, drug, circadian clock, and diet play important role in altering epigenetic modifications and in reducing vascular aging. ASC, factor–apoptosis-associated speck-like protein containing CARD, SMYD1, SET- and MYND- domain containing protein 1; SIRT, sirtuin; SCM-198, leonurine; β-HB, β-hydroxybutyrate. A detail of this figure is elaborated in section the epigenetic intervention strategies of vascular aging.

Evidence points to the possibility that structural and functional vascular aging can be reversed. Age-related declines in vascular function can be halted by lifestyle changes, such as exercise, diet, sleep, and genetic/epigenetic pharmacological intervention. These activities slow down age-related changes and the loss of functional blood vessels. A summary of epigenetic intervention mechanisms is shown in Figure 3.

### Health Lifestyle

#### Exercise

Regular physical activity improves vascular health and brings about discrete epigenetic modifications in tissues. For example, exercise increases the methylation levels of the pro-inflammatory factor apoptosis-associated speck-like protein containing CARD (ASC) gene (encoding IL-1β and IL-18), and thus suppress ASC-induced inflammation (130). Other evidence, shows that the histone methyltransferase, Smyd1, is protective roles in the pathological remodeling process (131), and that regular exercises increase SIRT1 expression and stimulates of NAD (+) biosynthesis (132). In addition, ubiquitination and phosphorylation have also been considered crucial regulatory mechanisms because they reduce protein misfolding, the accumulation of toxic proteins, and redundant “junk” proteins that occasionally accumulate in cells and tissues. Regular exercise stimulates ubiquitination and phosphorylation while promoting the elimination of defective proteins (133). MicroRNAs like miR-20a, miR-126, miR-210, and miR-221/222 reportedly regulating angiogenesis and maintaining vascular integrity and again the levels of these molecules are changed following exercise (134). Finally, exercise alters CTCF-based 3D chromatin architecture *via* an unknown mechanism (135).

#### Circadian Clock

The circadian clock significantly affects the expression of genes associated with obesity and type 2 diabetes. Poor circadian rhythm promotes hypomethylation in deleterious genes and hypermethylation in beneficial genes. This triggers an increase in transcriptional and genomic instability, chronic damage, and impacts on vascular homeostasis *viz*. the release of NO and growth hormone, MMP and ECM remodeling, and vascular stem cells niche. Circadian patterns also influence chromatin and promote self-repairing mechanisms like the activation of histone ubiquitination, and SIRT1 activation (136, 137).

#### Diet

Keeping a balanced and healthy diet delays vascular aging-related DNA methylation by regulating the methionine and S-adenosylmethionine cycle (95, 138). Caloric restriction is also thought to be a major factor in slowing down the aging processes of blood vessels (139).

### Drugs and Small Molecule Compounds

#### Renin-Angiotensin-Aldosterone-Systems (RAAS)

RAAS inhibits vascular remodeling and increases the expression of pro-survival genes like nicotinamide phosphoribosyltransferase, and SIRT3. This occurs *via* reducing mitochondrial oxidative stress, and highlights that the suppression of the RAAS system could be a possible way of managing vascular aging (140).

#### Statins

Statins have an ability to decrease DNA damage by inducing DNA damage repair systems and by suppressing oxidative stress by increasing antioxidant defenses (141). However, use of statins is often associated with adverse reactions like rhabdomyolysis that are of clinical significance. Here, we speculate leonurine (SCM-198), which is still in the clinical trial but shows fewer adverse reactions, will be a more promising drug in the future based on the present findings that it regulates H3K27 demethylase JMJD3 (142, 143). Moreover, the phase I clinical trial of 36 subjects uncover that SCM-198 affected homocysteine-methionine metabolism (144), which means that it can reduce ROS-induced endothelial cell damage and lipid peroxidation, thereby reversing vascular damage and destruction. The basic experimental studies also confirm its therapeutical effect (145, 146).

#### Metformin

A 78,241 people observational study has shown that the use of metformin increases life span (147). It is a known inducer of SIRT1, which increased SIRT1 promoter chromatin accessibility (148).

#### Spermidine

Spermidine prevents histone H3 hyperacetylation, can activate autophagy, and prolong life span (149). This anti-aging effect may be associated with its ability for restoring cellular metabolic dysfunction. A study of 85 elder adults reveals that spermidine significantly improved cognitive performance (150, 151).

#### Gut Microbiota

Long-term studies have found that gut microbiota disorders are related to malnutrition, obesity, diabetes, and diseases. ProBiotic-4, composed of mixture of *Bifidobacterium lactis, Lactobacillus casei, Bifidobacterium bifidum*, and *Lactobacillus acidophilus*, has been shown to attenuate aging-induced gut microbiota dysbiosis by reducing toll-like receptor 4 (TLR4) expression and influence nuclear factor-κB (NF-κB) nuclear translocation (152).

#### Other Small Molecule Compounds

Resveratrol is the only SIRT1 agonist that has been shown to delay vascular aging (110). Other molecules like, β-hydroxybutyrate (β-HB) have been found to prevent vascular senescence and can activate Oct4 expression level *via* stimulating DNA hydroxymethylation (153). Kallistatin attenuates TNF-α-induced endothelial progenitor cells (EPCs) senescence and STZ-induced aortic senescence by abolishing miR-34, and SIR-2.1 (154), Kallistatin is a candidate molecule that could be further developed as an epigenetic drug in the treatment of diabetes-induced vascular aging.

## Discussion and Perspectives

In summary, the following review has addressed the roles of epigenetic-mediated vascular aging, current discoveries, and possible roles of epigenetic processes in vascular aging. An understanding of these regulatory mechanisms critical in vascular aging will be paramount in developing novel treatment strategies. Epigenetic aging mechanisms that focus of much attention by researchers and growing evidence shows DNAm, post-translational histone modifications, ncRNAs, chromatin structure alteration are important in the cardiovascular system. In addition, several epigenetic-related drugs in clinical trials as highlighted by the WHO International Clinical Trial Registration Platform (WHO ICTRP). However, only two DNMTs inhibitors (azacytidine and decitabine), six HDACs inhibitors (vorinostat, romidepsin, belinostat, chidamide, panobinostat, and tazemetostat), and one ncRNA (siPCSK9) have been so far approved for clinical use. In the future, we are optimistic that other epigenetic based drugs will be developed. These developments arising from methodological improvements that allow for the analysis of epigenetic changes occurring at the single-cell and 3D level. These advances will make research easier and allow for studies addressing endothelial metabolism, angiogenesis, and pathological progress of arteriosclerosis during aging and collectively this will provide a dynamic pattern of change in cells and tissues. Most studies to date have stepped toward finding the role of 5mC reader (BAZ2A, MBD1/2, MBD4, MeCP2, SETDB1/2, UHRF1/2, and ZBTBR4/33/28), 5hmC and 5fmC readers (MHS6, PRP8, RPL26, UHRF2, EHMT1, FOXI3, FOXK1/2, FOXP1/4, L3MBTL2, MPG, and TDG), histone modification writer (EZH2, MMSET, DOT1L, SETD7, MLL1, PRMT1/3/5, and SMYD2/3), histone modification reader (CREBBP, EP200, MOZ, MOF, BRD2/3/4/7/9, and SMARCA2/4), histone modification eraser (HDAC4, JMJD3, and LSD1), and some ncRNAs in vascular aging. Many *in vitro and in vivo* studies have demonstrated some of these targets show the capacity in reducing vascular aging processes, but their pathologies and pharmacology are mostly unknown. Effects of these epigenetic targets that underpins the aging therapeutic should be considered in future studies.

## Author Contributions

YZ and ZL designed and revised the manuscript. QD, XL, YF, HH, and CH helped to improve the contexts and grammar. All authors contributed to the article and approved the submitted version.

## Funding

This work was supported by Macau Science and Technology Development fund [FDCT (0067/2018/A2, 033/2017/AMJ, 0007/2019/AKP, 0052/2020/A, 0011/2020/A1, and 0030/2018/A1)]. The National Natural Science Foundation of China (No. 81973320).

## Conflict of Interest

The authors declare that the research was conducted in the absence of any commercial or financial relationships that could be construed as a potential conflict of interest.

## Publisher's Note

All claims expressed in this article are solely those of the authors and do not necessarily represent those of their affiliated organizations, or those of the publisher, the editors and the reviewers. Any product that may be evaluated in this article, or claim that may be made by its manufacturer, is not guaranteed or endorsed by the publisher.
